# The evolution of mobile apps for asthma: an updated systematic assessment of content and tools

**DOI:** 10.1186/s12916-015-0303-x

**Published:** 2015-03-23

**Authors:** Kit Huckvale, Cecily Morrison, Jing Ouyang, Aseem Ghaghda, Josip Car

**Affiliations:** Global eHealth Unit, Imperial College London, Reynolds Building, St Dunstan’s Road, London, W6 8RP UK; Health Services and Outcomes Research Programme, Lee Kong Chian School of Medicine, Nanyang Technological University, Singapore, Singapore

**Keywords:** Android, App, Asthma, Cross-sectional study, iPhone, mHealth, Mobile health, Smartphone, Systematic assessment, Update

## Abstract

**Background:**

Interest in mobile apps that support long-term conditions such as asthma is matched by recognition of the importance of the quality and safety of apps intended for patient use. We assessed how changes over a 2-year period affected the clinical suitability of apps providing self-management information and tools for people with asthma by updating a review first performed in 2011.

**Methods:**

Systematic content assessment of all apps for iOS and Android examining the comprehensiveness of asthma information, consistency with the evidence base for asthma self-management and adherence to best practice principles for trustworthy content, comparing the quality of apps available in 2011 to those released since.

**Results:**

Between 2011 and 2013, numbers of asthma apps more than doubled from 93 to 191, despite withdrawal of 25% (n = 23/93) of existing apps. Newer apps were no more likely than those available in 2011 to include comprehensive information, such as the use of action plans, or offer guidance consistent with evidence; 13% (n = 19/147) of all apps, and 39% (n = 9/23) of those intended to manage acute asthma, recommended self-care procedures unsupported by evidence. Despite increases in the numbers of apps targeting specific skills, such as acute asthma management (n = 12 to 23) and inhaler technique (from n = 2 to 12), the proportion consistent with guidelines (17%, n = 4/23) and inhaler instructions (25%, n = 3/12), respectively, was low, and most apps provided only either basic information about asthma (50%, n = 75/147) or simple diary functions (24%, n = 36/147).

**Conclusions:**

In addition to persisting questions about clinical quality and safety, dynamic aspects of app turnover and feature evolution affect the suitability of asthma apps for use in routine care. The findings underline the need for coordinated quality assurance processes that can adapt to changing clinical and information governance-related risks, ensure compliance with the evidence base and reflect local variations in clinical practice. It is unclear if substantial clinical benefits can be realized from a landscape dominated by low quality generic information apps and tools that do not adhere to accepted medical practice.

**Electronic supplementary material:**

The online version of this article (doi:10.1186/s12916-015-0303-x) contains supplementary material, which is available to authorized users.

## Background

Medical apps for patients are software programs that run on personal mobile devices and offer functions such as disease tracking, access to clinical and peer support, health information, and reminders [1]. Survey data indicate that approximately three fifths of US and UK adults own an app-capable smartphone [2,3] and around half of adults report knowing about and using apps [2]. In 2012, a fifth reported having downloaded a health or fitness app, increasing amongst people with recent changes in health status [4]. Industry estimates suggest that, by 2018, over one billion individuals worldwide will use a health or fitness app [5] drawn from a pool of consumer health apps estimated to number in excess of 15,000 [6]. In 2014, over a third of US clinicians reported having recommended an app to a patient in the past year [7], and a survey of US adults suggested that nine of ten would use one issued ‘on prescription’ by a healthcare professional [8]. Among policymakers and clinicians, interest in apps reflects the potential to improve patient support and, potentially, health outcomes by equipping individuals with tools for self-management and remote monitoring [9-11]. However, there is also increasing recognition of the importance of ensuring that medical apps are effective [12,13], safe [14,15], meet patient needs [16,17], and comply with existing evidence-based clinical practice [18-20].

In 2011, we mapped [21] the types of apps available for patients with asthma, chosen as a prevalent long-term condition with potential social and economic [22,23] impacts and which requires a range of self-management skills in everyday life [24]. We explored the degree to which apps conformed to evidence-based recommendations derived from international asthma guidelines. We characterized the range of possible functions supported by apps for asthma, including information provision, peak flow and symptom diaries, and medication trackers. While highlighting the potential to support tailored education and assist clinical decision-making using monitoring data, the review uncovered a range of potential issues. Few information apps addressed key educational topics recommended in international guidance. Many advocated strategies for acute asthma management and trigger avoidance that were unsupported by current evidence. Assessment and diagnostic tools were largely unvalidated, some had errors of design, and the small number of apps offering therapeutic interventions had no basis in evidence.

Rapid evolution is characteristic of mobile technologies. New hardware and software capabilities create opportunities for health; for example, remote monitoring enabled by always-on network connections and wearable sensors [25]. Modern development methods mean that apps commonly undergo cycles of refinement in response to testing and user feedback [26]. Changes in mobile devices and software have been accompanied by a broadening discussion about quality and safety that has involved clinicians [27,28], policy groups [29], and, more recently, regulators [30,31]. Compared to 2011, there is greater understanding of potential risks and more resources targeted towards medical app developers, aiming to improve the quality of medical apps [32-35]. As a result, there is an opportunity to explore the impact that these changes may have had on apps intended for patient use.

We aimed to explore changes in the content, function, and clinical quality of apps since 2011. We used a systematic approach, based on a systematic literature review, to identify, classify, and review apps for asthma. By updating our earlier review, we sought to differentiate persistent quality issues from those that are new or have proven transient.

## Methods

### App selection

All apps from the two most widely-used [36] mobile platforms (Android and iOS) incorporated in the original review [21] were automatically eligible for inclusion. We chose not to include apps running on other platforms, such as Blackberry, whose market share is currently declining [36]. To identify new apps, we performed a stepwise process of search, screening, and review. We searched the public app stores of the two most popular smartphone operating systems using the terms ‘asthma’, ‘inhaler’, ‘peak flow’, and related word variants, e.g., ‘asthmatic’. After removing apps already included from the previous study, results were screened to eliminate obviously irrelevant content. The remaining apps were downloaded and reviewed to reach a final decision for inclusion. Screening and review were performed by three reviewers (KH, JO, and AG), working independently. Final decisions were reached by consensus.

Free and paid-for smartphone and tablet apps were eligible for inclusion if they contained asthma-specific content or tools intended for use by patients or the public (see list below). Apps targeted exclusively towards clinicians were not included. Apps were excluded if they could not be downloaded from UK app stores or, after at least two attempts on different test devices, would not start. Apps from any country were eligible provided they supported English.

#### Inclusion criteria

- Smartphone or tablet app
- Free or paid
- Running on Android or iOS platforms
- Content or tools addressing one or more aspects of asthma diagnosis, management, or support as either the sole function or in a way that means asthma-related elements can be isolated from the rest of the content
- Presenting content in any format
- English language
- Targeted at patients of any age

#### Exclusion criteria

- Not available through an approved device marketplace
- Explicitly disclaimed use for a health-related purpose
- Could not be downloaded because of country restrictions that prevented access in the UK
- Could not be used because of technical problems, after two attempts

### App assessment

Apps were assessed using a systematic approach to characterize functions and content. Apps containing information about asthma were evaluated using a set of criteria derived from US [37], UK [24], and international [38] asthma guidelines. Information content was evaluated for coverage of eight domains identified as priorities for self-management education (basic facts about asthma, basic principles of treatment, trigger avoidance, how to use treatment, self-monitoring skills, the role of action plans, managing exacerbations, and personalized care), using a set of operational criteria derived from guidelines. These criteria were those used in our earlier review except for one criterion in the self-monitoring domain which was modified in response to peer feedback to acknowledge symptom-based methods for self-monitoring (criteria and change detailed in Additional file 1: Table AF1). Coverage of each educational domain was rated as either complete (all criteria satisfied), partial (some criteria satisfied), or absent. Content was also evaluated for consistency with evidence-based recommendations concerning eight aspects of asthma management derived from guidelines (Additional file 1: Table AF2). For those apps with content addressing a particular topic, the advice given was rated as being either consistent or inconsistent with underlying evidence. Consistency was judged both on the basis of factual accuracy and appropriate qualification of advice to reflect either uncertainty or limitations in the applicability of evidence, such as recommendations relevant only to individuals with more severe asthma, in which case we expected any information to be appropriately qualified. Information content was evaluated regardless of the delivery medium. However, apps focusing only on complementary and alternative medicine, using the definition of the NIH National Center for Complementary and Alternative Medicine [39], were excluded from this analysis because there was no basis to expect that they should comply with guideline recommendations. For diaries and other self-management tools, the nature of inputs and outputs and methods for summarizing and sharing data were characterized. For all apps with calculator and questionnaire functions, we attempted to locate validation information in the form of an authoritative citation. Where validation information was found, the behaviour of the app in handling inputs and producing appropriate outputs was assessed. All apps were assessed for compliance with principles for ethical disclosure of authorship and data protection practices, using criteria previously adapted from those assessing online health information devised by the Health on the Net Foundation [40] (criteria in Additional file 1: Table AF3). We also recorded any software issues that were encountered during testing, using a schema devised during the first review to classify problems such as crashes or errors in the user interface (detailed in Additional file 1: Table AF4).

Apps included in the original review had previously been assessed using these criteria. To ensure scoring consistency, we conducted a process of calibration in which a random selection of 10 apps was re-assessed and the outcomes compared to previous judgments. Apps that had been updated between 2011 and 2013 were also reassessed. Apps were reviewed by an author of the original review (KH) and one of two additional reviewers (JO and AG), working independently. After reconciliation, the results of the assessment process were recorded, along with basic characteristics of included apps, in a structured data extraction form (template in Additional file 1: Table AF5) for analysis.

### *Post hoc* evaluation

In addition to the planned analyses described above, we performed a *post hoc* evaluation of inhaler technique guidance in response to the growing number of apps that include this function, using criteria derived from manufacturer guidance to assess key steps, such as inhaler preparation, necessary for effective drug delivery into the lungs (detailed in Additional file 1: Table AF6).

### Data analysis

Apps available on multiple platforms were grouped for subsequent analysis. When an app was available as multiple versions on the same platform, for example ‘demo’ and ‘full’ editions, the most fully featured version was used for analysis (irrespective of free/paid status). The decision to define the unit of analysis as the platform-independent app reflected learning from the original review concerning the possible risk of bias resulting from double counting some apps. In that review, we treated each app version separately because we anticipated different quality issues arising from the need to tailor each app according to the platform and version. However, the final results suggested that this was not, in general, the case. While in 2011 only a small number of apps were affected (n = 13), and sensitivity analysis undertaken during that analysis demonstrated minimal impact on results, by 2013 the number of duplicates was substantially larger (n = 77) with consequently greater scope for bias.

Descriptive statistics were used to summarize the types, functions, and quality characteristics of apps available in 2011, those released since 2011, and overall. When reporting comparisons between 2011 and 2013, data are always written in chronological order, e.g., for “(X vs. Y)”, X are data from 2011 and Y reflect data for apps released since 2011. We hypothesized that newer apps might have different quality characteristics arising from greater experience amongst developers and discourse in both medical and computer science literature emphasizing the importance of high-quality medical apps. To explore differences in the quality of information apps, we compared the proportion of educational domains completely, partially, or not satisfied, and the proportion of guidance consistent with the evidence base in those available in 2011 and those released since. We also compared the proportion of apps satisfying principles for ethical disclosures of information and the proportion of apps with specific software issues. Apart from these planned comparisons, we performed a small number of additional tests to compare differences in the proportion of paid-for apps, apps addressing complementary and alternative medicine, and the incorporation of features such as support details, in-app help, and social sharing features. These are marked as *post hoc* comparisons in the results.

For all comparisons, we used a two-tailed Fisher’s exact test [41], with the Freeman-Halton extension [42] to handle two by three cases for information comprehensiveness and (*post hoc*) content orientation. To adjust for multiple comparisons, we used a Holm-Bonferroni [43] sequential adjustment to control the group-wise error rate at 5%, instead of specifying a fixed significance level for comparisons. All analyses were performed using R (Version 3.1.2).

## Results

**Figure 1:**
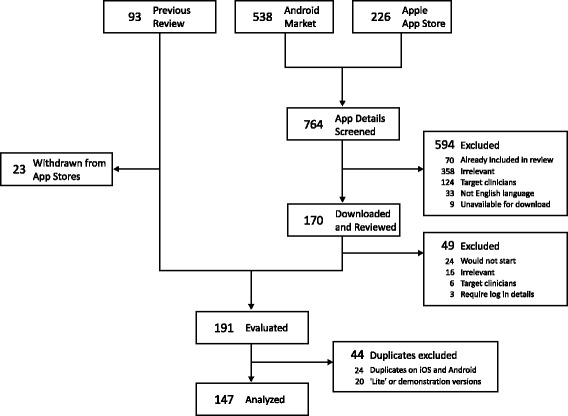
**Flowchart of app selection process.** Flowchart details the process of selecting individual apps for inclusion in the study. After evaluation of 191 apps, duplicates available on both iOS and Android (n = 24) and, when more fully featured versions were available, ‘lite’ or demonstration versions (n = 20) were excluded, leaving 147 unique apps for subsequent analysis.

Searches performed in June, July, and August 2013 identified 764 apps (Figure 1, n = 538 Android, 226 Apple). After excluding unsuitable apps (n = 643), 191 apps were selected for evaluation. These included 70 apps identified in the 2011 review that remained available. Details of apps excluded at the final review stage are provided in Additional file 2: Table AF7.

**Figure 2:**
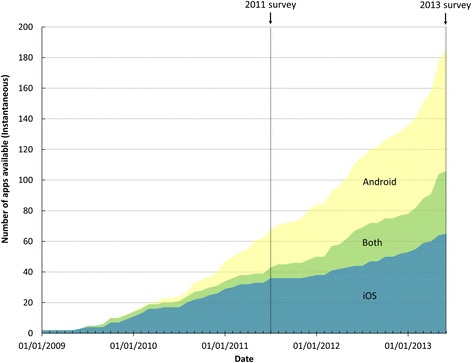
**Growth in numbers of asthma apps.** Plot shows the total number of available apps for asthma against time, based on the original release date, for the period 2009 to mid-2013. Withdrawals are accounted for. Vertical lines indicate the dates of the 2011 and 2013 surveys. Data were not available for 5 apps; therefore, final n = 186.

The number of apps for asthma more than doubled between 2011 and 2013 (from 93 to 191), dominated by growth in apps available for Android (from 41 to 103, 151% increase, vs. iOS 52 to 88, 69% increase, charted over time in Figure 2). Forty-one apps were available on both Android and iOS platforms and a further 36 had multiple versions on a single platform. After removing 24 platform duplicates and 20 demonstration versions where a more fully-featured version was available, analysis was performed on 147 unique apps. Over a quarter (28%, n = 22/78 unique apps) included in the original review had been withdrawn, and a similar proportion (24%, n = 19/78) updated, between 2011 and 2013. Withdrawals are included in reported 2011 statistics but not in overall 2013 totals. At the conclusion of analysis in June 2014, a further 20 apps had been withdrawn. These are retained for analysis, but annotated as withdrawn in data tables provided in Additional file 2.

Table 1 summarizes the basic characteristics of included apps. Compared to apps available in 2011, apps released since were significantly more likely to be free (33%, n = 26/78 vs. 64%, n = 59/92, *P* <0.001, *post hoc*) but also more likely to carry advertising (18%, n = 14/78 vs. 34%, n = 31/92, *P* = 0.024, *post hoc*), although the difference was not significant after sequential adjustment. Between 2011 and 2013 the median cost of paid-for apps decreased from £1.27 GBP to £0.89 (£1.12 overall), reflecting a reduction in the proportion of apps charging more than £1.00 (37%, n = 29/78 vs. 22%, n = 20/92). The proportion of apps offering in-app upgrades to unlock addition functions remained unchanged at 9% (n = 13/147) overall, (8%, n = 7/78 vs. 7%, n = 6/92, *P* = 0.576, *post hoc*). While the US remained the main source of apps for asthma, accounting for almost half of apps with a known country of origin available in 2013 (48%, n = 52/108), the number of countries represented increased from 13 to 21. Although the overall proportion of apps indicating that the product was targeted at a particular group remained low (16%, n = 24/147), apps released since 2011 were significantly more likely to provide information targeting parents/caregivers (4%, n = 3/78 vs. 16%, n = 15/92, *P* = 0.011, *post hoc*). There was also a non-significant increase in the proportion of apps targeting children (3%, n = 2/78 vs. 8%, n = 7/92, *P* = 0.182, *post hoc*).

**Table 1:** **Basic characteristics of included apps**

	**2011 (n = 78)**		**New 2013 (n = 92)** **¤**		**All 2013 (n = 147)** **¤**	
**Characteristic**	**Yes**	**%**	**Yes**	**%**	**Yes**	**%**
**Platform**						
Android only	32	41%	52	57%	73	50%
iOS only	40	51%	28	30%	57	39%
Both	6	8%	12	13%	17	12%
**Cost**						
Free	26	33%	59	64%	80	54%
Paid-for	52	67%	33	36%	67	46%
Median	£1.27	–	£0.89	–	£1.12	–
**Country of origin** ^**†**^						
US	24	31%	31	34%	52	35%
UK	5	6%	6	7%	8	5%
India	7	9%	7	8%	8	5%
Australia	3	4%	4	4%	7	5%
Other	10	13%	24	26%	33	22%
Unknown	29	37%	20	22%	39	27%
**Content orientation**						
Conventional medicine only	56	72%	59	64%	101	69%
Complementary/alternative medicine only	15	19%	17	18%	26	18%
Mixed	7	9%	16	17%	20	14%
**Audience targeting** ^**‡**^						
Children or young adults	2	3%	7	8%	8	5%
Parents or caregivers	3	4%	15	16%	19	13%
No audience stated	73	94%	73	79%	123	84%
**Other features**						
Adverts	14	18%	31	34%	39	27%
Social sharing	13	17%	35	38%	61	41%
Cloud storage	3	4%	10	11%	16	11%
Clinical disclaimer	32	41%	37	40%	55	37%
In-app help	14	18%	28	30%	43	29%
Online help	5	6%	6	7%	10	7%

¤ New 2013, Apps available in 2013 that were not available in 2011; All 2013, All apps available in 2013, excluding those available in 2011 that were subsequently discontinued.

^†^Four countries with highest overall number of apps in 2013 shown. Other countries were: Ireland (n = 5), Germany (n = 4), Switzerland (n = 4), Japan (n = 3), Romania (n = 2), Singapore (n = 2), Austria (n = 1), France (n = 1), Italy (n = 1), Norway (n = 1), Poland (n = 1), Portugal (n = 1), Spain (n = 1), and Sweden (n = 1).

^‡^Audience targeting was identified by specific claims made in the app title or description or content. We found no cases where apps explicitly indicated they were intended for adult use only. A small number of apps targeted both children and a parent or caregiver (2011, n = 0; New 2013, n = 3; All 2013, n = 3).

Information provision remained the function most commonly offered by nearly three fifths of apps for asthma (56%, n = 83/147; Table 2), followed by self-management tools in almost half (48%, n = 70/147). The number of apps combining self-management tools, such as diaries, with any form of information content grew from one (1%) in 2011 to nine (6%) in 2013. As in 2011, small numbers of apps available in 2013 provided functions for assessing asthma symptoms using questionnaires (n = 11) or sensors (n = 2) or assisting with aspects of therapy (n = 6). Although there was an increase in the proportion of apps released since 2011 incorporating mixed content addressing both conventional and alternative medical traditions, the change in relative frequencies was not significant (*P* = 0.268, *post hoc*; Table 2) and the overall proportion of apps presenting content oriented towards conventional medical practice changed minimally (69% n = 101/147 vs. 73% n = 57/78 in 2011).

**Table 2:** **Breakdown of app functions**

	**By date** ^**†**^						**By platform**	
	**2011 (n = 78)**		**New 2013 (n = 92)** **¤**		**All 2013 (n = 147)** **¤**		**All 2013 (n = 191)**	
**Function**	**n**	**%**	**n**	**%**	**n**	**%**	**Android**	**iOS**
**Information**	37	47%	60	65%	83	56%	66	44
General information	36	46%	51	55%	74	50%	56	39
Acute asthma management (First aid)	12	15%	18	20%	23	16%	19	14
Inhaler technique guidance	2	3%	10	11%	12	8%	6	13
Other therapeutic instructions	13	17%	10	11%	16	11%	15	9
**Self-management tools**	36	46%	38	41%	70	48%	42	49
Diaries and trackers	21	27%	18	20%	36	24%	23	26
Pollen status	3	4%	8	9%	12	8%	6	10
Pollution status	3	4%	6	7%	9	6%	2	9
Allergen database	4	5%	3	3%	7	5%	5	4
Forum	0	0%	1	1%	1	1%	1	0
Online pharmacy	0	0%	1	1%	1	1%	1	0
Combined with information	1	1%	8	9%	9	6%	6	7
**Assessment tools**	9	12%	8	9%	16	11%	10	11
Physiological measurement	2	3%	1	1%	3	2%	0	4
Assessment questionnaires	4	5%	7	8%	11	7%	7	9
Standalone calculators	2	3%	2	2%	4	3%	3	1
**Therapeutic tools**	2	3%	5	5%	6	4%	1	5

The table compares the types of functions present in apps for asthma available in 2011 and 2013, and provides a breakdown by platform for 2013; 25 apps included in the 2011 assessment were withdrawn and this accounts for the lower overall total in 2013. Some apps contained multiple functions and are counted more than once.

^†^Apps available on both Android and iOS are counted only once.

¤ New 2013, Apps available in 2013 that were not available in 2011; All 2013, All apps available in 2013, excluding those available in 2011 that were subsequently discontinued.

### Information apps

The number of apps offering information about asthma increased from 37 in 2011 to 83 by 2013, with 60 new apps and 14 withdrawals. Most information apps available in 2013 incorporated general information about asthma (89%, n = 74/83), although a growing proportion of apps (23%, n = 18/78 vs. 34%, n = 31/92, *P* = 0.174, *post hoc*) also incorporated specific instructions about the management of acute asthma, inhaler technique, and other aspects of self-management (discussed further in Therapeutic Instructions and Tools, below.) Apps used a range of media to communicate information including text (64%, n = 53/83), video and animation (10%, n = 8/83), or by combining different media types (27%, n = 22/83). A significantly greater proportion of apps released since 2011 used a medium other than text to communicate information (11%, n = 4/37 vs. 42%, n = 25/60, *P* = 0.001, *post hoc*). A new development was the use of gameplay (n = 3) to provide interactive education in three products targeted at children and teenagers. Full details of information-containing apps are provided in Additional file 2: Table AF8.

Although increasing in number, the proportion of products presenting information based exclusively around conventional medical practices was unchanged (51%, n = 19/37 vs. 50%, n = 30/60). Just over a fifth of all apps available in 2013 (23%, n = 19/83) combined alternative and conventional information and the remainder (28%, n = 23/83) presented information based on alternative medical principles alone. Consequently, there were 59 apps (vs. 23 in 2011) incorporating conventional medical information for which it was possible to assess coverage of self-management education topics and consistency with guideline recommendations.

Over two thirds (83%, n = 49/59) of all apps available in 2013 provided at least some details about basic aspects of asthma pathophysiology (Table 3). However, only just over half provided information about basic principles of medical management (53%, n = 31/59), and fewer addressed key self-management skills, including allergen and trigger avoidance (44%, n = 26/59), self-monitoring skills (39%, n = 23/59), or how to use treatment appropriately (39%, n = 23/59). Less than a third of apps addressed how to recognize the signs of deterioration (31%, n = 18/59), the importance of personalized treatment and goal setting (31%, n = 18/59), and the role of an action plan (22%, n = 13/59). There was no significant difference in the proportion of apps released since 2011 addressing domains other than basic facts (27%, n = 6/22 vs. 20%, n = 9/45, *P* = 0.542), nor in the proportion of apps addressing each domain considered individually (*P* values shown in Table 3). While the overall number of products addressing three or more educational domains, at least partially, grew from 14 to 31 between 2011 and 2013, new apps were not more likely to address multiple domains (64%, n = 14/22 vs. 51% n = 23/45, *P* = 0.435). In the same period, the number of products addressing all eight domains at least partially grew from two to five (with one withdrawal). As in 2011, however, no product provided complete coverage of all eight domains by satisfying all criteria.

**Table 3:** **Coverage of asthma self-management education topics by information apps**

	**2011 (n = 23)**				**New 2013 (n = 44)** **¤**					**All 2013 (n = 59)** **¤**			
**Educational topic**	**No**	**Partially**	**Wholly**	**%** ^**†**^	**No**	**Partially**	**Wholly**	**%**	***P*** **value** ^**‡**^	**No**	**Partially**	**Wholly**	**%**
Basic facts about the nature of the condition	2	15	6	91%	9	26	9	80%	0.510	10	37	12	83%
Allergen and trigger avoidance	9	12	2	61%	25	19	0	43%	0.094	33	24	2	44%
The nature of treatment: relievers and preventers	11	7	5	52%	22	18	4	50%	0.333	28	25	6	53%
Recognizing and responding appropriately to acute exacerbations	14	7	2	39%	30	9	5	32%	0.728	41	12	6	31%
How to use treatment	16	6	1	30%	27	17	0	39%	0.259	36	22	1	39%
Self-monitoring and assessment skills	16	6	1	30%	27	16	1	39%	0.632	36	20	3	39%
The role of a written, personalized action plan	18	2	3	22%	34	5	5	23%	1.000	46	6	7	22%
Personalizing the definition of good asthma control	17	5	1	26%	29	15	0	34%	0.244	41	18	0	31%

The table compares the proportion of apps containing asthma self-management education information that address guideline-recommended topics either partially or wholly between 2011 and 2013. Apps focusing solely on complementary and alternative medicine were excluded.

^†^The proportion of information apps that wholly or partially address a given educational topic.

^‡^Two-tailed probability obtained from the Freeman-Halton extension to Fisher’s exact test of the comparison of topic coverage in apps available in 2011 and those released subsequently. After applying a Holm-Bonferroni correction to control the family-wise error rate at less than or equal to 5%, no comparisons achieved significance.

¤ New 2013, Apps available in 2013 that were not available in 2011; All 2013, All apps available in 2013, excluding those available in 2011 that were subsequently discontinued.

Thirty-six apps (vs. 14 in 2011) were evaluated for consistency of recommendations against nine evidence-based topics (Table 4) because they contained content addressing at least one topic. Smoking cessation and passive smoking avoidance were the topics most commonly addressed by apps (by n = 19 and 21, respectively) and, as in 2011, were correctly recommended as beneficial by all apps. All apps addressing immunotherapy (n = 5) and weight reduction (n = 7) correctly advocated their use in specific patient populations. However, apps were also likely to recommend actions for which there is unclear evidence of benefit, such as mold (n = 12/12) and cockroach (n = 7/7) avoidance measures, removal of pets (n = 7/8), and avoidance of air pollution (n = 15/17). Most apps covering seasonal influenza vaccination recommended it (n = 8/10) without acknowledging the uncertain impact on asthma [44]. Compared to 2011, there were no significant differences in the proportion of apps released since that presented advice consistent with the evidence base, either when considering each statement individually (exact *P* values computed from proportions shown in Table 4 were all 1.0) or when comparing the proportion of apps containing no statements contradicting evidence (36%, n = 5/14 vs. 43%, n = 12/28, *P* = 0.747). A new finding in 2012 concerned a small number apps (n = 6) containing advice emphasizing the possible harmful effects of conventional medicine and, in two cases, promoting a commercial product, for example a homeopathic remedy. Previously, apps containing mixed content had tended to emphasize the right to choose between medical approaches rather than denying accepted medical practice.

**Table 4:** **Consistency of information apps with evidence-based recommendations**

	**2011**			**New 2013** **¤**			**All 2013** **¤**		
**Statement**	**n** ^**†**^	**Consistent**	**%** ^**‡**^	**n**	**Consistent**	**%**	**n**	**Consistent**	**%**
Removal of pets from the home (Qualified benefit)	3	1	33%	7	1	14%	8	1	13%
Fungal allergen avoidance and control measures (Qualified benefit)	8	0	0%	9	0	0%	13	0	0%
Cockroach avoidance and control measures (Qualified benefit)	6	0	0%	4	0	0%	7	0	0%
Cessation of active smoking (Beneficial)	7	7	100%	14	14	100%	19	19	100%
Avoidance of passive smoking (Beneficial)	8	8	100%	15	15	100%	21	21	100%
Avoidance of exposure to air pollution (Qualified benefit)	8	1	13%	14	1	7%	17	2	12%
Immunotherapy in atopic asthma (Beneficial)	1	1	100%	4	4	100%	5	5	100%
Weight reduction in obese patients (Beneficial)	3	3	100%	4	4	100%	7	7	100%
Seasonal influenza vaccination (Qualified benefit)**	4	1	25%	8	2	25%	10	2	20%

The table compares the proportion of apps containing particular management advice that is consistent with evidence-based recommendations between 2011 and 2013. The expected advice is shown in parentheses. We use the term ‘qualified benefit’ when additional factors, such as personal sensitivity to particular allergens or aspects of personal choice, are relevant concerns and unconditional recommendations are therefore inappropriate. Apps focusing solely on complementary and alternative medicine were excluded. Statistical comparison between apps in 2011 and new apps in 2013 was performed but is not shown because all comparisons, performed using Fisher’s exact test, yielded an exact probability of 1.0.

^†^The number of apps that took a stance on a given topic, for example, by stating that a given action is definitely effective in controlling asthma symptoms.

^‡^The proportion of apps whose stance is consistent with the evidence base.

¤ New 2013, Apps available in 2013 that were not available in 2011; All 2013, All apps available in 2013, excluding those available in 2011 that were subsequently discontinued.

**Seasonal influenza vaccination is routinely offered to asthmatic patients; however, it is unclear if vaccination reduces the severity or frequency of flu-related asthma exacerbations [44].

### Diaries and trackers

Thirty six apps available in 2013 (24%, vs. 27% in 2011) were diaries for recording asthma symptoms (n = 27), peak flow measurements (n = 24), and medication (n = 24, detailed in Additional file 2: Table AF9). Six apps allowed a user to document an action plan. Six incorporated inhaler trackers, including two where this was the sole function, designed to alert a user when a metered dose inhaler or other medication was running low. One app connected to an electronic inhaler to automatically log doses. Two thirds of diaries (67%, n = 24/36) offered a mechanism to summarize either recorded symptoms (n = 4), peak flow (n = 11), or both (n = 9) in chart form. Over half (55% n = 11/20) of apps charting peak flow used predicted (n = 6), personal best (n = 7), or manually entered (n = 3) thresholds to classify charted data to aid interpretation, for example, as ‘green’, ‘yellow’, and ‘red’ zones. Nine apps provided alerts based on changing input values. Seven provided specific advice, either by excerpting relevant parts of a user supplied-action plan (n = 3/6 apps with action plan support) or displaying standardized text (n = 4). Sixteen allowed reminders to be set to take peak flow measurements (n = 9), medication (n = 3), appointments (n = 2), and vaccination (n = 1). Compared to 2011, there were increases in the proportion of apps synchronizing and storing data online (10%, n = 2/21 vs. 44%, n = 8/18, *P* = 0.025, *post hoc*) but not those offering a mechanism for sharing data with clinicians (62%, n = 13/21 vs. 61%, n = 11/18, *P* = 1.000, *post hoc*), for example, email or file export.

### Other self-management tools

Nineteen apps (compared to 6 in 2011, Table 2) provided status information about pollen (n = 10), air pollution (n = 7), or both (n = 2; Additional file 2: Table AF10). Most (n = 12) provided coverage of locations in the US. Only one product, which relied on used-supplied data, offered global coverage. Seven apps (vs. 4 in 2011) offered databases of chemical additives to allow patients to identify potential triggers in food and cosmetic ingredients. One app combined an electronic version of an action plan with links to information about asthma. Another product connected to a forum where users could view topics about asthma management and post their own questions. A third app offered UK patients the ability to purchase asthma medication online.

### Diagnostic and assessment tools

Fifteen apps (vs. 6 in 2011) contained diagnostic or assessment functions. Ten products offered questionnaires for asthma diagnosis (n = 4) or assessment (n = 6; Additional file 2: Table AF11). Two apps used the validated Asthma Control Test under license and one app reused a questionnaire developed by the University of Maryland. The remaining apps either did not provide a citation or validation information or the information provided was insufficient to verify the basis for assessment. Nine products provided predicted peak flow calculators as either the main function (n = 3) or integrated into a diary (n = 6). No calculator app provided a clear citation, but the underlying formula was identified for two apps. Issues first identified in 2011 resulting in incorrect calculation output for two apps where the formula was deduced were unresolved. Three products used the device microphone to attempt to quantify lung volume (n = 1) and analyse breath sounds (n = 2), but were either unvalidated or experimental products that had not yet received regulatory approval.

### Therapeutic instructions and tools

Forty-one apps (28% of n = 147) contained specific instructions for the use of inhalers (8%, n = 12/147), first aid for acute exacerbations (16%, n = 23/147), or other approaches to the management of asthma such as the use of herbal remedies (11%, n = 16/147, breakdown in Additional file 2: Table AF12). After pressurized metered dose inhalers (pMDI), either alone (100%, n = 12/12) or with spacer devices (92%, n = 11/12), the devices most commonly addressed by inhaler technique guidance were Accuhalers (58%, n = 7/12) and Turbohalers (50%, n = 6/12), although a range of additional devices were also covered (Additional file 2: Table AF12). Apps used text (33%, n = 4/12), video (25%, n = 3/12), animation (8%, n = 1/12), or a combination (33%, n = 4/12) to provide guidance. *Post hoc* evaluation (criteria in Additional file 2: Table AF6) examined the proportion of apps providing complete and correct information about inhaler preparation (67%, n = 8/12), positioning in the mouth (67%, n = 8/12), and the sequence of inhalation and actuation (50%, n = 6/12). While a quarter (25%, n = 3/12) addressed all three domains, the majority of apps omitted or misstated at least one step necessary for effective delivery of drugs to the lungs [45,46] for at least one of the inhalers discussed. The most common error (in 25%, n = 4/12) was the failure to explain how inspiration should start before, and continue after, actuation of a pMDI. A variable proportion of apps addressed other aspects of inhaler use such as holding the breath after inhalation (83%, n = 10/12), timing of a second dose (58%, n = 7/12), and mouth rinsing to reduce the risk of candidiasis with inhaled steroids (50%, n = 6/12). While five apps (42%) provided information about identifying empty Turbohalers or Accuhalers, no app addressed dose-tracking using pMDI. The two apps (17%) that covered inhaler care both provided incorrect information about spacer cleaning that risked static build-up interfering with subsequent doses.

Of the 23 apps incorporating advice about the management of acute asthma, approximately half (52%, n = 12/23) provided a clear description of the signs of acute asthma and gave practical advice such as sitting a patient up (48%, n = 11/23) and offering reassurance (52%, n = 12/23). Three fifths (61%, n = 14/23) provided appropriate instructions about when to seek further help. While almost two thirds (65%, n = 15/23) recommended the use of a rescue inhaler, less than a third (30%, n = 7/23) provided specific dose and timing instructions and only four apps (17%) provided instructions appropriate for children in addition to adults. Overall, four apps (17%) addressed all five components (Additional file 2: Table AF12). The proportion of apps released since 2011 that made treatment recommendations based on alternative medical practices did not change significantly (76%, n = 13/17 vs. 56%, n = 14/25, *P* = 0.207, *post hoc*), and almost three fifths of apps available in 2013 (58%, n = 19/33), and 39% (n = 9/23) of those intended to manage acute asthma, recommended procedures unsupported by evidence, for example, the use of a hot water bottle to warm the chest, cider vinegar administered orally, or fasting for the duration of an attack.

A small number of products (n = 6 vs. 2 in 2011) were tools intended to have a therapeutic effect. Three apps provided training in aspects of Buteyko breathing, which may have a future role in asthma management [47]. The remaining three were based on alternative medical principles using hypnosis, mantra, and motivational messages.

### Disclosures and software issues

Results of assessment for compliance with ethical disclosures about authorship and data protection are summarized in Table 5 (based on criteria detailed in Additional file 1: Table AF4). Perhaps reflecting the increase in apps syncing data to a cloud service (4%, n = 3/78 vs. 11%, n = 10/92, *P* = 0.145, *post hoc*) or offer social sharing (17%, n = 13/78 vs. 38%, n = 35/92, *P* = 0.002, *post hoc*), apps released since 2011 were significantly more likely to have a privacy policy (9%, n = 7/78 vs. 36%, n = 33/92, *P* <0.001). New apps were also significantly more likely to provide contact details (41%, n = 32/78 vs. 64%, n = 59/92, *P* = 0.003). While no apps in 2011 had an editorial or advertising policy, five new apps did. In general, apps provided limited information about the information sources used for the advice they contained. The proportion of apps providing any kind of content attribution was unchanged (36%, n = 21/78 vs. 33%, n = 26/92, *P* = 0.856). Almost three fifths of information-containing apps (57%, n = 47/83) available in 2013 repackaged content available freely online from resources such as Wikipedia, a small increase from 2011 (54%, n = 19/35). The majority (77%, n = 36/47) of these apps did so without referencing the original content sources. Overall, 35% (n = 29/83) were ‘junk’ apps that reused, without attribution, information of generally low quality that was freely available online in a paid-for or advert-supported format.

**Table 5:** **Summary of ethical disclosures and software issues**

	**2011 (n = 78)**		**New 2013 (n = 92)** **¤**			**All 2013 (n = 147)** **¤**	
**Quality domain**	**Yes**	**%** ^**†**^	**Yes**	**%**	***P*** **value** ^**‡**^	**Yes**	**%**
**Ethical disclosures**							
Attribution of authorship^§^	21	36%	26	33%	0.856	40	33%
Purpose clearly stated	69	88%	88	96%	0.090	141	96%
Privacy policy available	7	9%	33	36%	<0.001*	44	30%
Information versioning	1	1%	3	3%	0.626	4	3%
Contact details provided	32	41%	59	64%	0.003*	90	61%
Funding model clear	21	27%	30	33%	0.502	49	33%
Editorial/advertising policy	0	0%	5	5%	0.063	5	3%
**Software issues**							
Data entry validation issues**	9	33%	8	32%	1.000	16	33%
Functionality issues	13	17%	9	10%	0.252	15	10%
User interface issues	25	32%	34	37%	0.522	45	31%
Crashes	8	10%	8	9%	0.796	13	9%
Network issues	3	4%	20	22%	<0.001*	25	17%
Other issues	10	13%	18	20%	0.301	28	19%

The table compares the proportion of apps satisfying best practices for content attribution and ethical disclosures (detailed in Additional file 1: Table AF3), and those with software issues identified during testing (detailed with examples in Additional file 1: Table AF4), between 2011 and 2013.

^†^The proportion of information apps that satisfy a particular domain. Unless otherwise stated, the denominator is the total number of apps shown in the relevant heading.

^‡^Two-tailed probability obtained from Fisher’s exact test of the comparison of topic coverage in apps available in 2011 and those available since 2013. *After applying a Holm-Bonferroni correction to control the family-wise error rate within each domain at less than or equal to 5%, three comparisons were significant.

^§^Denominator for proportions reflects only apps with attributable content, e.g., written information, measurement scales, pollen/pollution data. Attribution of the software itself lies with the developers whose details are always released. Denominators: 2011 n = 59, New 2013 n = 80, 2013 n = 120.

¤ New 2013, Apps available in 2013 that were not available in 2011; All 2013, All apps available in 2013, excluding those available in 2011 that were subsequently discontinued.

**Data entry validation concerns steps taken to prevent out-of-range or inappropriate data being stored in an app, for example, textual values allowed in a numeric field. As a result, the denominator reflects only apps permitting data entry, e.g., calculators, diaries. Denominators: 2011 n = 27, New 2013 n = 25, 2013 n = 49.

We also assessed software issues encountered during the test process (using criteria detailed in Additional file 1: Table AF5). Compared to 2011, there was no change in the proportion of new apps lacking appropriate data entry validation to prevent, for example, textual data being entered in numeric data fields (33%, n = 9/27 vs. 32%, n = 8/25, *P* = 1.000). The proportion of apps with problems with the user interface, such as mislabelled data fields (32%, n = 25/78 vs. 37%, n = 34/92, *P* = 0.522), or core functionality, such as the correct operation of a calculator (17%, n = 13/78 vs. 10%, n = 9/92, *P* = 0.252), was also unchanged. However, there was a significant increase in apps released since 2011 experiencing network problems when trying to access online content or services (4%, n = 3/78 vs. 22%, n = 20/92, *P* <0.001). Crashes affected about one in ten apps during testing, unchanged from 2011 (9%, n = 13/147 overall).

## Discussion

Our updated findings present a mixed picture for clinicians interested in integrating apps into routine care. While choice has increased, newer apps for asthma were no more likely than those available in 2011 to satisfy evidence-based recommendations for information content or the design of self-management tools. Our data extend findings of low overall compliance with evidence in recent assessments of medical apps for disease-management [20,48] and health-promotion [49,50] by suggesting that issues of quality are sustained and are not isolated to first-generation apps. However, as in 2011, our results question the scope for benefit offered by current apps for asthma. Firstly, rather than focusing on known gaps in self-management where there is evidence that intervention can improve outcomes, such as the use of action plans [22,51], most apps offer either very basic background information about asthma or simple peak flow diaries. Only a fifth of information apps available in 2013 addressed action plans and the proportion of diaries offering a mechanism for recording a plan was smaller. Secondly, although it is recognized that information provision alone has a limited impact on health behaviour [52], the proportion of apps combining information and tools remains small. While apps using multimedia methods, such as video and gameplay, presented welcome new opportunities for tailored support that focuses on specific needs, such as improving inhaler technique [53], or on specific groups, such as those with limited literacy [54], our assessment highlights the risk of clinically-relevant impacts resulting from inadequate content. For example, three quarters of inhaler training apps left out at least one step considered necessary for effective delivery of drug into the lungs. Thirdly, while apps incorporate a range of features, such as reminders, that could plausibly play a role in interventions designed to improve asthma self-management, apps themselves do not currently provide effective scaffolding to support individuals in changing, and sustaining, behaviour. For example, although many apps offered the ability to send data by email, and the proportion of apps using a mechanism to share data online almost doubled between 2011 and 2013 to 52%, no app offered a built-in mechanism for communication between a professional and patient, potentially limiting the scope for feedback that could assist self-management. Nor was it clear that the online services linked to currently available apps could support the kind of summary and flagging activities necessary to manage cohorts rather than single patients. Evidence is needed that app-supported remote monitoring can generate reliable data, prompt consistent and sustained clinical improvements, and meet the needs of patients across the spectrum of asthma severity [55]. Although more apps allowed information to be shared using a social network like Facebook or Twitter, this was never in the context of a structured activity relating to asthma self-management and no apps took advantage of the possibilities for peer support. Similar to the findings of others [19,56], only one asthma app appeared to be linked to an active research project [57], highlighting a broader disconnect between guidance on the design of interventions that incorporate evidence-based components and evaluation [58] and what occurs in practice.

Dynamic aspects of the asthma app marketplace, including rapid growth and turnover, have potential consequences for both clinical services and individuals. Over a quarter of apps included in the original review, including those from reputable content providers, had been withdrawn. While patients may choose apps to suit their changing needs, the costs associated with app selection, staff training, setting up support mechanisms, and appropriate governance create incentives for planned care to be based around small numbers of trusted apps. Unexpected withdrawal of an app may be an inconvenience for individual users, but can threaten the function of an established clinical service. App updates are also potentially disruptive, requiring testing and training. As a result, formal arrangements with app developers, such as service-level contracts, should be considered where apps are being used as part of planned care. Systematic identification of factors influencing app lifespan would be helpful and future work should address this.

A new finding concerned apps that repackaged existing information either to create content focused on promoting specific commercial products, or to serve as a more general vehicle for hosting adverts operating on a pay-per-click basis. A small number specifically emphasized negative effects associated with conventional medical practice, with the intention of promoting commercial alternatives. Some apps were developed using online tools that specifically target developers with no or limited coding experience. Marketing materials for these tools emphasized their ability to produce apps quickly and in number. If the goal of these apps is simply to generate advertising revenue or an initial purchase, content need only to appear superficially plausible. This kind of low-quality information, coupled with high volume distribution and commercial motive, shares similarities with spam or junk email and websites [59]. Indeed, some apps for asthma reused material originally produced by online content farms [60]. If sustained, the junk medical app would be an unwelcome development, making it harder for users to locate higher-quality and unbiased content. Unlike the web, where search engines tend to prioritize higher-quality content in results [61], conventional app stores offer limited mechanisms for filtering out lower quality and junk apps.

Apps offering unvalidated diagnostic, assessment, and therapeutic functions with no basis in evidence are a continuing issue that raises questions about patient safety and clinical liability [62]. While some apps are likely to fall under the scope of medical app regulation recently proposed in both the US and Europe [30,31], aspects of how this will work in practice are yet to be agreed, not least because regulators must balance competing demands of risk, costs, and the possible impact on innovation [14]. As a result, rapid action appears unlikely, even where standards already exist. For example, while physical sensors, such as peak flow meters, are classified as moderate risk (class 2) medical devices, neither of the two asthma apps that use a device microphone to analyse breath sounds appear to have undergone regulatory approval, and both remain available in November 2014, over 1 year since guidance first came out. Regulation is also constrained by the scope of current legislation which applies only to apps intended to diagnose, treat, or prevent disease. While not ruling out future changes, regulators have stated that information apps do not satisfy this definition, even if they contain procedural guidance such as first aid [63], and that they intend to apply discretion when enforcing rules for apps that probably qualify as medical devices but pose a low overall risk such as diaries, pollen alerts, and inhaler trackers [14]. While ensuring that potentially useful apps are not burdened by costly and time-consuming approvals, some apps will fall outside established reporting mechanisms that would allow a more complete picture of risk to emerge over time. Further work is necessary to understand the consequences of known defects in tools such as predicted peak flow calculators and identify unanticipated risks, for example, those arising from novel services like online pharmacies that do not involve a usual clinician.

Our findings have relevance for emerging medical app accreditation programs [32,64,65], which aim to reduce the burden of assessment placed on professionals and patients, provide recognizable badges of app quality, and collate certified apps to make them easily accessible. Rapid app turnover and feature evolution suggest that accreditation processes will need to be revised regularly and programs will need to allocate appropriate resources to reassess their growing cohort of existing certified apps as they are updated. Programs must also consider the possibility of best practices that vary by location and target group. For example, some US inhaler technique guidance apps advised actuating a metered dose inhaler in front of an open mouth, a method not taught in UK self-management education. The growth in connected apps, and those based around advertiser models, highlights the importance of assessment processes which examine privacy and security-related risks as personal and medical data start to be shared online. While we did not explore the governance arrangements of cloud-based apps, privacy and security are topical concerns for patients [55,66]. Additionally, recent findings highlighting gaps in privacy disclosures [67,68] and security arrangements [69,70] in popular health apps suggest that further scrutiny is required. High rates of app turnover emphasize the importance of due-diligence that focuses on the commitment of developers to maintaining their products. The range of functions seen within a single long-term condition in this study highlights the potential complexity of defining comprehensive assessment standards. Similar to the content-independent criteria applied in this study, simplified assessments which focus on high-risk functions and proxy markers for quality, such as the involvement of clinicians in design, have been proposed [71,72], but their predictive validity is unknown. While most accreditation programs appear to be developing their own criteria, a collaborative, open approach to standard development could share the workload, for example, by involving professional bodies with specific content expertise, enable transparent input by patients, professionals, and developers, and permit evaluation. Further work should explore the extent to which developers can be engaged proactively to increase awareness of accreditation requirements and the potential value of patient and professional input into health app design, which, in turn, may increase the likelihood that apps meet accreditation requirements without potentially costly cycles of rejection and resubmission. Finally, because accreditation programs are voluntary and have yet to prove their ability to control quality issues at scale, most health apps available in public app stores will not be badged for quality. Patient education could address this by, for example, explaining the phenomenon of junk medical apps and characterizing the risks as similar to those presented by the range of information available on the general internet.

The study used a systematic method to exhaustively sample apps from the two most popular app stores and included steps to minimize bias during the assessment process. By repeating an assessment first performed in 2011, we were able to explore aspects of the dynamics of app turnover and feature development. However, the study had a number of limitations. Apps in languages other than English and those available on platforms other than Android and iOS were not included. It is possible that there are systematic differences in app quality by language or platform that this study could not detect. Because the study involved only two time points, there should be substantial caution in inferring that any changes observed are indicative of trends. Our data reflect issues present in apps available at particular points in time, and while representative categories are likely to persist, the specifics will change, necessitating updated assessment. Finally, because of limitations in the data provided by app stores, we were unable to precisely quantify the scale of app use by patients nor the ways in which apps are used. The potential for a mismatch between available features and those desired by patients was highlighted by a recent review of apps for patients with Parkinson’s disease [73]. Future work could explore this issue in relation to asthma, examine whether user reviews and ratings have any predictive validity for app quality, and continue to develop the currently limited evidence-base specifically addressing the design of apps for asthma self-management [74].

## Conclusions

In addition to established findings about variable clinical quality and possible safety risks, dynamic aspects of the asthma app marketplace, such as rapid growth and turnover, affect the suitability of apps for use in clinical settings. Efforts to assure the quality of medical apps need to be responsive to the changing features and risks posed by apps, consider governance as well as clinical factors, and recognize the differing needs of patients using apps on their own initiative and those supported by clinical care. Accreditation programs may need to be supplemented by proactive intervention by regulators to address potentially unsafe apps and advice to help patients navigate the increasing numbers of poor quality or ‘junk’ apps for asthma in general app stores.

## Additional files

Additional file 1:
**Tables AF1–6.** Contains: **Table AF1.** Operational criteria for assessing the comprehensiveness of asthma self-management educational materials. Criteria covering eight key educational domains, derived from UK British Thoracic Society/Scottish Intercollegiate Guideline Network (BTS/SIGN), US Expert Panel Report 3 (EPR-3), and Global Initiative for Asthma (GINA) guidelines. **Table AF2.** Management strategies used to assess consistency of asthma information with evidence-base. Strategies and evidence-base derived from UK BTS/SIGN, US EPR-3, and GINA guidelines. **Table AF3.** Ethical disclosure principles for smartphone apps. Adapted from the Health on The Net Foundation principles for health information on the internet. **Table AF4.** Categories of software issue considered during assessment. Categories used to group any software errors, user interface problems, or other issue encountered during testing. **Table AF5.** Data extraction template. Template into which details of apps were recorded for subsequent analysis. **Table AF6.** Operational criteria for assessing inhaler technique education. Criteria covering eight domains, derived from product manufacturer guidance and UK BTS/SIGN, US EPR-3, and GINA guidelines. Additional file 2:
**Tables AF7–12.** Contains: **Table AF7.** Characteristics of excluded apps. Table detailing apps excluded from analysis and the reasons for exclusion. **Table AF8.** Characteristics of information apps. Table of apps containing written or multimedia information about asthma. Detailed results of evaluation for consistency and comprehensiveness with evidence-based guidelines and adherence to best-practices for content development disclosures. **Table AF9.** Characteristics of diary apps. Table of apps containing asthma diary and related tracking functions. Details of diary function types, action plan support, adherence to best-practices for content development disclosures, and any issues identified during testing. **Table AF10.** Characteristics of apps with other self-management functions. Table of apps containing self-management functions other than diaries and trackers such as air pollution information. Details of function types, adherence to best-practices for content development disclosures, and any issues identified during testing. **Table AF11.** Characteristics of apps with assessment functions. Table of apps containing calculator and questionnaire functions related to asthma diagnosis or management. Details of function types, validation information (if identified), and adherence to best-practices for content development disclosures and any issues identified during testing. **Table AF12.** Characteristics of apps with therapeutic instructions and functions. Table of apps with an intended therapeutic purpose. Details of function types, validation information (if identified), adherence to best-practices for content development disclosures, and any issues identified during testing.
