# Supplementary material for: The evolution of mobile apps for asthma: an updated systematic assessment of content and tools
Source: BMC Med. 2015 Mar 23;13:58. doi: 10.1186/s12916-015-0303-x (PMC4391129; doi:10.1186/s12916-015-0303-x)
Supplement: Additional file 1: — Tables AF1–6. Contains: Table AF1. Operational criteria for assessing the comprehensiveness of asthma self-management educational materials. Criteria covering eight key educational domains, derived from UK British Thoracic Society/Scottish Intercollegiate Guideline Network (BTS/SIGN), US Expert Panel Report 3 (EPR-3), and Global Initiative for Asthma (GINA) guidelines. Table AF2. Management strategies used to assess consistency of asthma information with evidence-base. Strategies and evidence-base derived from UK BTS/SIGN, US EPR-3, and GINA guidelines. Table AF3. Ethical disclosure principles for smartphone apps. Adapted from the Health on The Net Foundation principles for health information on the internet. Table AF4. Categories of software issue considered during assessment. Categories used to group any software errors, user interface problems, or other issue encountered during testing. Table AF5. Data extraction template. Template into which details of apps were recorded for subsequent analysis. Table AF6. Operational criteria for assessing inhaler technique education. Criteria covering eight domains, derived from product manufacturer guidance and UK BTS/SIGN, US EPR-3, and GINA guidelines. [file 12916_2015_303_MOESM1_ESM.pdf]

Apps for Asthma – Additional File 1 – Tables AF1-6.pdf

Portable Document Format

- Table AF1

  Operational criteria for assessing the comprehensiveness of asthma self-management educational materials

  Criteria covering eight key educational domains, derived from UK BTS/SIGN, US EPR-3 and GINA guidelines.

- Table AF2

  Management strategies used to assess consistency of asthma information with evidence-base

  Strategies and evidence-base derived from UK BTS/SIGN, US EPR-3 and GINA guidelines.

- Table AF3

  Ethical disclosure principles for smartphone apps

  Adapted from the Health on The Net Foundation principles for health information on the internet.

- Table AF4

  Categories of software issue considered during assessment

  Categories used to group any software errors, user interface problems of other issue encountered during testing.

- Table AF5

  Data extraction template

  Template into which details of apps were recorded for subsequent analysis.

- Table AF6

  Operational criteria for assessing inhaler technique education

  Criteria covering eight domains, derived from product manufacturer guidance and UK BTS/SIGN, US EPR-3 and GINA guidelines used for a post-hoc evaluation.

- References

Table AF1

Operational criteria for assessing the comprehensiveness of asthma self-management educational materials

Domains and criteria are based on UK BTS/SIGN[1], US EPR-3[2] and GINA[3] guidelines and were used in our earlier review[4]. In response to peer feedback, we modified one criterion under the self-monitoring domain. Reflecting the validity of both peak and symptom-based approaches to monitoring, we removed the statement: “All patients with asthma should have a peak flow meter,” and replaced it with “States that both symptom recognition and peak flow measurements are valid approaches for self-monitoring.” Both UK and GINA guidelines had been updated (updated versions are cited here), however the criteria were not otherwise affected by the change.

**Domain**	**Criteria**
Basic facts about the nature of the condition	▪ States that asthma is a lung disease characterized by inflammation and narrowing of the airways;<br>▪ States that the four main symptoms of asthma are cough, wheeze, shortness of breath and chest tightness;<br>▪ States that asthma cannot be cured (although childhood symptoms may remit) but can be effectively controlled;<br>▪ States that the cause of asthma is not known.
The nature of treatment: relievers and preventers	▪ States that there are two classes of medication: relievers and preventers;<br>▪ Explains possible side effects of medication (tachycardia/tremor in B_2 agonists; thrush/cataracts/dysphonia for inhaled steroids; possible additional effects for high dose steroids)<br>▪ States that early treatment can prevent symptoms from worsening.
Allergen and trigger avoidance	▪ States that recognizing and avoiding personal triggers is an important part of asthma control;<br>▪ Provides guidance consistent with the primary and secondary prophylaxis components of the BTS/SIGN guidelines in relation to specific triggers.
How to use treatment	▪ States that preventer medication must be used regularly to be effective;<br>▪ States the importance of good inhaler technique and appropriate use of a spacer device[1]<br>▪ States the importance of ensuring inhalers are in date and are not empty.
Self-monitoring and assessment skills	▪ States that learning to recognize signs of change in asthma status is an important personal skill;<br>▪ States that both symptom recognition and peak flow measurements are valid approaches for self-monitoring.<br>▪ Explains the purpose of a peak flow meter and how to use it;<br>▪ States the importance of regular physician review.
The role of a written, personalized action plan	▪ States that patients with asthma should have an up to date written action plan;<br>▪ Explains the purpose of an action plan (to step up and step down treatment, and to seek appropriate help in response to changing symptoms and/or peak flow).

Domain	Criteria
Recognizing and responding appropriately to acute exacerbations	▪ Describes signs/symptoms of worsening asthma (increasing wheeze; cough; night time disturbance; breathlessness limiting activity; reliever inhalers not working);<br>▪ States the importance of changing treatment and/or seeking help promptly.<br>▪ Steps involved in lay management of acute asthma<br>o Recognizing the signs of an asthma attack<br>o Appropriate rescue inhaler use<br>o Other practical steps, e.g. sitting the patient up<br>o Providing reassurance<br>o When to seek emergency assistance
Personalizing the definition of good asthma control	▪ States that it is reasonable for most people to achieve minimal symptoms and limitation of activities;<br>▪ Asks patients to reflect on what they would consider as good asthma control;<br>▪ Advocates discussion with personal health provider to set treatment goals in partnership.

[1] Because of the complexity of addressing a range of different inhaler types, inhaler technique is addressed separately, see Table AF2.

Table AF2

Management strategies used to assess consistency of asthma information with evidence-base.

Strategies were extracted from UK BTS/SIGN[1], US EPR-3[2] and GINA[3] guidelines. Criteria are those developed for our earlier review[4]. We considered only management strategies discussed by all three sets of guidelines. 'Qualified benefit' was used where factors were identified that should shape the scope of the evidence, such as evidence applicable only to individuals with severe asthma; where a strategy might be considered for reasons other than those directly relating to asthma, as in the case of seasonal influenza vaccination; or where the advice given by the guidelines differed.

Management strategy	Evidence-base	Guideline positions
Removal of pets from the home	Qualified benefit	[1] "**The reported effects of removal of pets from homes are paradoxical**, with either no benefit for asthma, or a potential for continued high exposure to induce a degree of tolerance."<br>[2] [EPR-3 suggests that animal removal **could be considered** but rates the evidence Grade D, which reflects panel consensus only]<br>[3] "Complete avoidance of pet allergens is impossible […] Although removal of such animals from the home is encouraged, even after permanent removal of the animal it can be many months before allergen levels decrease and **the clinical effectiveness of this and other interventions remains unproven**."
Fungal allergen avoidance and control measures	Qualified benefit	[1] "Although fungal exposure has been strongly associated with hospitalization and increased mortality in asthma, **no controlled trials have addressed the efficacy of reduction of fungal exposure** in relation to control of asthma."<br>[2] "The Expert Panel recommends consideration of measures to control indoor mold […] but **the relative contribution of fungi, house-dust mites or irritants [to asthma symptoms] is not clear**."<br>[3] "Air conditioners and dehumidifiers may be used to reduce humidity to levels less than 50% and to filter large fungal spore. **However, air conditioning and sealing of windows have also been associated with increases** in fungal and house dust mite allergens."

Management strategy	Evidence-base	Guideline positions
Cockroach avoidance and control measures	Qualified benefit	[1] "Cockroach allergy is not a common problem in the UK and studies of attempts to avoid this allergen elsewhere have **produced conflicting results**."<br>[2] [EPR-3 recommends cockroach control **if the patient is sensitive to cockroaches**]<br>[3] "[Measures for cockroach control] are **only partially effective in removing residual allergens**."
Cessation of active smoking	Beneficial	[1] "**Direct** or passive exposure to cigarette smoke **adversely affects** quality of life, lung function, need for rescue medications for acute episodes of asthma and long term control with inhaled steroids."<br>[2] "[Smoke exposure] is associated with **increased symptoms, decreased lung function, and greater use of health services** among those who have asthma."<br>[3] "Asthma patients who smoke and are not treated with inhaled glucocorticosteroids, have a **greater decline in lung function** than asthmatic patients who do not smoke."
Avoidance of passive smoking	Beneficial	[1] "Direct or **passive** exposure to cigarette smoke **adversely affects** quality of life, lung function, need for rescue medications for acute episodes of asthma and long term control with inhaled steroids."<br>[2] "[Smoke exposure] is associated with **increased symptoms, decreased lung function, and greater use of health services** among those who have asthma."<br>[3] "Secondhand smoke **increases the frequency and severity of symptoms** in children with asthma."
Avoidance of exposure to air pollution	Qualified benefit	[1] "Time-series studies suggest that **air pollution may provoke** acute asthma attacks or aggravate existing chronic asthma although the effects are **very much less than those with infection or allergen exposure**."<br>[2] "Clinicians [should] advise patients to avoid, to the extent possible, exertion or exercise outside when levels of air pollution are high."<br>[3] "Avoidance of unfavorable environmental conditions is **usually unnecessary** for patients whose asthma is controlled."

**Management strategy**	**Evidence-base**	**Guideline positions**
Immunotherapy for a defined allergen	Beneficial	[1] "Immunotherapy can be considered in patients with asthma where a **clinically significant allergen cannot be avoided**."<br>[2] "Immunotherapy [should] be considered for patients who have persistent asthma if evidence is clear of a **relationship between symptoms and exposure to an allergen** to which the patient is sensitive."<br>[3] "Appropriate immunotherapy requires the **identification and use of a single well-defined clinically relevant allergen**."
Weight reduction in obese patients	Beneficial	[1] "One randomized parallel group study has shown **improved asthma control following weight reduction** in obese patients with asthma."<br>[2] "Obesity has been associated with **asthma persistence and severity** in both children and adults. […W]eight loss in adults resulted in improvement in pulmonary mechanics, improved FEV1, reductions in exacerbations and courses of oral corticosteroids and improved quality of life."<br>[3] "Weight reduction in obese patients with asthma […] has been demonstrated to **improve lung function, symptoms, morbidity and health status**."
Seasonal influenza vaccination	Qualified benefit	[1] "Immunizations should be administered **independent of any considerations related to asthma**."<br>[2] "[We recommend] that **clinicians consider inactivated influenza vaccination for patients who have asthma** […] however the vaccine should **not be given with the expectation that it will reduce either the frequency or severity of asthma** exacerbations during the influenza season."<br>[3] "Patients with moderate to severe asthma **should be advised to receive an influenza vaccination** every year […] **however routine influenza vaccination** of children and adults with asthma **does not appear to protect them from asthma exacerbations or improve asthma control**."

Table AF3

Ethical disclosure principles for smartphone apps

Adapted from the Health on The Net Foundation principles for health information on the internet[16] and developed for our earlier review[4].

#	**Principle**
1	Information must be authoritative: all medical information presented by [and/or calculations performed by an app] must be attributed to an author and his/her training in the field must be mentioned.
2	Purpose [of the app]: A statement clearly declaring that the [app] is not meant to replace the advice of a health professional has to be provided. A brief description of the [app]'s mission, purpose and intended audience is necessary. Another brief description of the organization behind the [app], its mission and its purpose is also necessary.
3	Confidentiality: The [app publisher] must describe its privacy policy regarding how you treat confidential, private or semi-private information such as email addresses and the content of emails received from or sent to [its users]
4	Information must be documented, referenced and dated: All medical content [including calculations and formulae] has to have a specific date of creation and a last modification date.
5	Justification of claims: All information about the benefits or performance of any treatment (medical and/or surgical), commercial product or service are considered as claims. All claims have to be backed up with scientific evidence (medical journals, reports or others).
6	[App] contact details: The [app] must be operational and the information must be accessible and clearly presented. There must be a way to contact the [app publisher], such as a working email address or contact form, for visitors who would like to have more details or support.
7	Funding: [The app publisher] must include a statement declaring its sources of funding.
8	Editorial and advertising policy: Conflicts of interest and external influences which could affect the objectivity of the editorial content must be clearly stated in the disclaimer. All [apps] displaying paying banners have to have an advertising policy. This policy must explain how the [publisher] distinguishes between editorial and advertising content and which advertisements are accepted. Any conflict of interest has to be explained.

Table AF4

Categories of software issue considered during assessment

Categories used to group any software errors, user interface problems of other issue encountered during testing, extending those identified by our original review [4].

Issue type	Description	Example(s)
Data entry validation	▪ Data can be entered that are out-of-range or inappropriate, for example, textual values in a numeric field.<br>▪ New data can overwrite existing data without warning.	▪ Negative values of peak flow can be entered and are stored.<br>▪ New entries can overwrite existing data without warning.
Functionality	▪ A function of the app (e.g. calculation, data saving) does not operate as expected.	▪ App miscalculates the score of Asthma Control Test for adult.<br>▪ App displays an 'unfortunately you did not beat your highest score' message even if score is 100%.
Presentation and user interface (UI)	▪ Content having spelling and layout mistakes.<br>▪ User interface controls (e.g. textboxes, labels, buttons) are mislabeled, inoperative or inaccessible.<br>▪ Navigation between different parts of the app does not occur as expected or can lead to the user getting stuck on a particular screen.	▪ Some controls hidden when opened on a lower resolution screen.<br>▪ Text box for recording peak flow labelled as 'Peek Flow'.<br>▪ Some user controls not labelled in English.
Crash	▪ App stops responding in a timely way to user input or was closed unexpectedly by the smartphone or tablet operating system.	▪ App crashes when a backup of data entered by the user is attempted.
Network	▪ Problems with online or other data services (e.g. a website that an app downloads data from).	▪ Content does not load as expected.<br>▪ Synchronization with online service fails.
Other	▪ Any other software issue not described above.	▪ GPS function does not work.

Table AF5

Data extraction template

Template into which details of apps were recorded for subsequent analysis.

#	Field	Format
0.1a	App ID	Number
0.1b	Product ID	Number
0.1c	Linked IDs	Comma-separated numbers
0.2	Date of latest review	Date
0.3	Reviewer	Text
0.4	Downloaded	Yes/No
0.5	Platform	Pick
0.6a	Include or Exclude?	Include/Exclude
0.6b	If 0.6a EXCLUDE, provide reason	Text
0.7	Brief description	Text
0.8	Taxonomy	Text
Basic details		
1.1a	App name	Text
1.1b	URL	Text
1.1c	Display name	Text
1.1d	Namespace	Text
1.1e	Bundle ID	Text
1.2	Version	Number
1.3a	Initial release date	Date
1.3b	Version release date	Date
1.3c	Latest Version	Number
1.3d	Latest version release date	Date
1.3e	Number of version releases	Number
1.4	Cost	£ or 'Free'
1.5a	Publisher	Text
1.5b	Publisher ID	Number
1.6	Source country	Text
1.7a	Publisher website	Link
1.7b	Website accessible?	Yes/No
1.7c	Website spoofed?	Yes/No
1.7d	Actual website	Link
1.7e	Published email	Email Address
1.8a	Original number of downloads	Number or Number Range
1.8b	Original download count census date	Date
18.c	Updated number of downloads	Number or Number Range
1.8d	Updated download count census date	Date
1.8e	Original user rating average	Number
1.8f	Original user rating count	Number
1.8g	Original user rating census date	Date
1.8h	Updated user rating average	Number
1.8i	Updated user rating count	Number
1.8j	Updated user rating census date	Date
1.8k	Content rating	Text
1.9a	Languages	Yes/No
1.9b	Is UI translated?	Yes/No
1.9c	Is content translated?	Yes/No

#	Field	Format
Functionality		
2.0	App description	Text
2.1a	Main function 1	Text
2.1b	Main function 2	Text
2.1c	Main function 3	Text
2.3a	Is the app intervention standalone?	Yes/No
2.3b	If 2.4a NO, provide details of intervention	Text
2.4	Is there a user setup wizard?	Yes/No
2.5	Does the app support multiple user profiles?	Yes/No
2.6a	Does the app require network access?	Pick from list
2.6b	Can the app request location information (GPS/WiFi/Cell triangulation)?	Pick from list
2.6c	Can the app request access to read contact data?	Pick from list
2.6d	Can the app request access to read calendar data?	Pick from list
2.6e	Can the app access the camera/images?	Pick from list
2.6f	Can the app access the microphone?	Pick from list
2.6g	Can the app set up Bluetooth connections?	Pick from list
2.6h	Can the app set up NFC connections?	Pick from list
2.6j	Can the app send or receive data by IR?	Pick from list
2.6j	Can the app start automatically (excluding notifications)?	Pick from list
2.6k	Does the app use push or local notifications?	Pick from list
2.6l	Can the app place phone calls?	Pick from list
2.6m	Can the app send SMS or MMS using native functions?	Pick from list
2.6n	Can the app modify/delete storage outside its sandbox?	Pick from list
2.6o	Can the app read the phone status or identifiers?	Pick from list
2.6p	Can the app read calls or SMS or other message content?	Pick from list
2.6q	Can the app prevent the device from sleep?	Pick from list
2.6r	Can the app modify global settings?	Pick from list
2.6s	Can the app read sync or account settings?	Pick from list
2.6t	Can the app modify WiFi settings?	Pick from list
2.6u	Can the app terminate or restart other apps?	Pick from list
2.6v	Can the app list other running apps?	Pick from list
2.6w	Can the app set preferred status for one or more purposes?	Pick from list
2.6x	Can the app control device vibration?	Pick from list
2.7	Is the app purpose clear from the product advert?	Yes/No
Diary features		
3.1	Does the app have diary, questionnaire or diagnostic/assessment features?	Yes/No
If 3.1 NO, skip to the section 4		
3.2a	Does the app have a diary to record peak flow readings (PEFR)?	Yes/No
3.2b	If 3.2a YES, provide details	Text
3.2c	If 3.2a YES, does the app use a predicted or % of PB to classify readings?	Predicted/PB/Both/No
3.2d	If 3.2c PREDICTED, what formula is used to calculate predicted PEFR?	Text or 'Unknown'
3.2e	If 3.2a YES, is there a PEFR summary visualization?	Yes/No
3.2f	If 3.2e YES, provide details	Text
3.3a	Does the app have a diary to record symptoms or asthma-related events?	Yes/No
3.3b	If 3.3a YES, provide details	Text

#	Field	Format
3.3c	If 3.3a YES, is there a symptom summary visualization?	Yes/No
3.3d	If 3.3c YES, provide details	Text
3.4a	Does the app have a diary to record medication?	Yes/No
3.5a	If 3.2a or 3.3a YES, does the app provide any alert based on changing PEFR/symptoms?	Yes/No
3.5b	If 3.2a or 3.3a YES, does the app provide any advice based on changing PEFR/symptoms?	Yes/No
3.5c	IF 3.5b YES, what advice is given?	Text
3.6a	Does the app include any diagnostic or assessment tools, questionnaires or calculators?	Yes/No
3.6b	If 3.6a YES, provide details	Text
3.6c	If 3.6a YES, provide sources	Text
3.6d	If 3.5a YES, are sources clear?	Yes/No
3.6e	If 3.6a YES, what is the purpose of the assessment questionnaire/calculator?	Pick from list
3.7a	Does the app allow data to be shared with a clinician?	Yes/No
3.7b	If 3.7a YES, provide details	Text
Alerts		
4.1a	Does the app provide pollen information?	Yes/No
4.1b	Does the app provide pollution information?	Yes/No
4.2a	If 4.1a or 4.1b YES, where are the data sourced from?	Text
4.2b	If 4.1a or 4.1b YES, are sources clear?	Yes/No
4.2c	If 4.1a or 4.1b YES, what areas are covered?	Text
4.2d	If 4.1a or 4.1b YES, is the information dated?	Yes/No
4.3a	Does the app provide any reminders or notifications?	Yes/No
4.3b	If 4.3a YES, provide details	Text
Information		
5.1	Does the app provide any information or guidance about asthma?	Yes/No
5.2	Are information sources attributed?	Yes/No
5.2a	Provide details of attribution (or failure of attribution)	Text
5.2b	Third-party sources	Text
5.2c	Is the content reproduced from free online resource(s) other than those of the publisher?	Yes/No
5.3a	Is information content dated?	Date
5.3b	Is a content expiration date set?	Date
Action plan		
6.1a	Does the app recommend the use of a personal asthma action plan?	Yes/No
6.1b	Does the app have a feature to store personal asthma triggers?	Pick from list
6.1c	Does the app have a feature to store a personal asthma action plan?	Yes/No
6.2a	Does the plan use a 3+1 stage template?	Yes/No
6.2b	If 6.2a NO, how does it differ?	Text
6.3a	Does the app use the plan to make treatment recommendations?	Yes/No
6.3b	If 6.3a YES, provide details	Text
Asthma education information domains		
Does the app…		

#	Field	Format
7.1a	State that asthma is a lung disease characterized by inflammation and narrowing of the airways.	Yes/No
7.1b	State that the four main symptoms of asthma are cough, wheeze, shortness of breath and chest tightness.	Yes/No
7.1c	State that asthma cannot be cured (although childhood symptoms may remit) but can be effectively controlled.	Yes/No
7.1d	State that the causes of asthma are multifactorial/not well understood (triggers defined below)	Yes/No
7.2a	Explain the basis of asthma treatment as a partnership between patient and caregiver in which the aim is guided self-management.	Yes/No
7.2b	State that partnership requires investment and will develop over time.	Yes/No
7.2c	Emphasize the importance of sharing information, expectations, fears and concerns with care providers.	Yes/No
7.2d	Explain the role of joint setting of realistic goals for asthma control and management.	Yes/No
7.3a	State that there are two classes of asthma medication: relievers and preventers.	Yes/No
7.3b	Explain possible side effects of medication (tachycardia/tremor in B2 agonists; thrush/cataracts/dysphonia for inhaled steroids; possible additional effects for high dose steroids).	Yes/No
7.3c	State that early treatment can prevent symptoms from worsening.	Yes/No
7.4a	State that recognizing and avoiding personal triggers is an important part of asthma control.	Yes/No
7.5a	State that preventer medication must be used regularly to be effective.	Yes/No
7.6b	State the importance of good inhaler technique (or demonstrates) and appropriate use of a spacer device.	Yes/No
7.6c	State the importance of ensuring inhalers are in date and are not empty.	Yes/No
7.7a	State that learning to recognize signs of change in asthma status is an important personal skill.	Yes/No
7.7b	States that both symptom recognition and peak flow measurements are valid approaches for self-monitoring.	Yes/No
7.7c	Explain the purpose of a peak flow meter and how to use it.	Yes/No
7.7d	State the importance of regular physician review.	Yes/No
7.8a	State that patients with asthma should have an up to date written action plan.	Yes/No
7.8b	Explain the purpose of an action plan (to step up and step down treatment, and to seek appropriate help in response to changing symptoms and/or peak flow).	Yes/No
7.9a	Describe signs/symptoms of worsening asthma (increasing wheeze; cough; night time disturbance; breathlessness limiting activity; reliever in halers not working).	Yes/No
7.9b	State the importance of changing treatment and/or seeking help promptly.	Yes/No
7.9c	Explain the steps involved in lay management of acute asthma.	Yes/No

#	Field	Format
7.10a	State that it is reasonable for most people to achieve minimal symptoms and limitation of activities.	Yes/No
7.10b	Ask patients to reflect on what they would consider as good asthma control.	Yes/No
7.11	Notes	Text
Evidence base compliance		
What stance does the app take on the following issues…		
8.1	The use of dust mite allergen control measures.	Pick from list
8.2	The removal of pets from the home to improve asthma.	Pick from list
8.3	The use of fungal allergen avoidance and control measures.	Pick from list
8.4	The use of cockroach allergen avoidance and control measures	Pick from list
8.5	Smoking cessation.	Pick from list
8.6	The avoidance of passive smoking.	Pick from list
8.7	The avoidance of exposure to outdoor air pollution.	Pick from list
8.8	The avoidance of a defined food allergen, once identified.	Pick from list
8.9	Immunotherapy for a define allergen to control asthma	Pick from list
8.10	Weight reduction in obese individuals as a way of controlling asthma.	Pick from list
8.11	Seasonal influenza vaccination in people with asthma.	Pick from list
8.12a	Dietary supplementation with selenium to improve asthma.	Pick from list
8.12b	Low sodium diet to improve asthma.	Pick from list
8.12c	Spinal manipulation to treat asthma.	Pick from list
8.12d	Homeopathic medicine to treat asthma.	Pick from list
8.12e	Yoga to improve asthma.	Pick from list
8.12f	Breathing techniques including Buteyko and Papworth methods to improve asthma.	Pick from list
8.12g	Relaxation techniques or hypnosis to treat asthma.	Pick from list
8.12h	Acupressure to treat asthma	Pick from list
8.12i	Acupuncture to treat asthma	Pick from list
8.12j	Herbal or Chinese medicine to treat asthma	Pick from list
8.13	Notes	Text
Emergencies		
9.1	Does the app provide advice on what to do during an asthma attack?	Yes/No
If 9.1 NO, skip to section 10		
9.2	If 9.1 YES, who is the guidance targeted towards?	Pick from List
9.3a	If 9.1 YES, does the advice explain what the symptoms of an asthma attack are (cough, wheeze, shortness of breath, chest tightness)?	Yes/No
9.3b	If 9.1 YES, does the advice address how to use a reliever inhaler appropriately?	Yes/No
9.3c	If 9.1 YES, does the advice include sitting patient up and encouraging to take slow steady breaths.	Yes/No
9.3d	If 9.1 YES, does the advice include staying calm and offering reassurance.	Yes/No
9.3e	If 9.1 YES, does the advice include seeking emergency help if no improvement or any concerns (blue finger tips, quiet breath sounds)	Yes/No
9.4	Notes	Text
Inhaler technique guidance		

#	Field	Format
10.1	Does the app provide guidance on how to user an inhaler?	If Yes, choose which inhalers. Otherwise 'No"
If 10.1 NO, skip to section 11		
10.2a	If 10.1 YES, what medium is used to communication guidance?	Text/Video/Animation
10.3a	If 10.1 YES, does the guidance correctly address inhaler preparation?	Correct/Incorrect/Not Addressed
10.3b	If 10.1 YES, does the guidance correctly address positioning?	Correct/Incorrect/Not Addressed
10.3c	If 10.1 YES, does the guidance correctly address the inhalation/actuation method?	Correct/Incorrect/Not Addressed
10.3d	If 10.1 YES, does the guidance correctly address exhalation sequence?	Correct/Incorrect/Not Addressed
10.3e	If 10.1 YES, does the guidance correctly address secondary doses?	Correct/Incorrect/Not Addressed
10.4a	If 10.1 YES, does the guidance correctly address mouth rinsing?	Correct/Incorrect/Not Addressed
10.4b	If 10.1 YES, does the guidance correctly address ensuring inhaler not empty?	Correct/Incorrect/Not Addressed
10.5	If 10.1 YES, does the guidance correctly address inhaler care?	Correct/Incorrect/Not Addressed
10.6	Notes	
Information and security governance		
11.1a	Does the app offer password or PIN protection?	Yes/No
11.1b	Can entered data be edited?	Yes/No
11.1c	Can entered data be individually deleted?	Yes/No
11.1d	Can all stored data be wiped (other than through uninstallation)?	Yes/No
11.2a	Does the app require a user account to be created?	Yes/No
11.2b	If 11.2a YES, provide details	Text
11.3a	Does the app offer cloud sync?	Yes/No
11.3b	Does the app offer a backup or data export mechanism (apart from cloud sync and/or backup services provided by OS)	Yes/No
11.3c	If 11.3b YES, detail the export mechanism.	Pick from list
11.3d	If 11.3b YES, is the export encrypted?	Yes/No
11.3e	If 11.3b YES, provide details	Text
11.3f	Does the app offer social sharing features?	Yes/No
11.3g	If 11.3f YES, provide details	Text
11.4a	Does the app include or link to a privacy policy or confidentiality policy?	Pick from list
11.4b	If 11.4a YES, what is the URL for the policy?	Link
11.4c	If 11.4a YES, does the URL work?	Yes/No
11.4d	If 11.4a YES, are there any disclosures in the privacy policy that are surprising/unusual?	Text
11.4e	Does the marketplace or supplier website include a privacy policy?	Yes/No
11.4f	If 11.4e YES, what is the URL for the policy?	Link
11.4g	if 11.4e YES, does the URL work?	Yes/No
11.4h	If 11.4e YES, are there any disclosures in the privacy policy that are surprising/unusual?	Text

#	Field	Format
11.5a	Can the app transmit data to a third party (other than advertisers or user-initiated actions)?	Yes/No
11.5b	If 11.5a YES, provide details	Text
Content and attribution		
12.1a	What is the app publishers funding model?	Pick from list
12.1b	Is the app publisher also the commissioner of the app?	Yes/No/Unclear
12.1c	If 12.1b NO, who commissioned the app?	Text
12.1d	If 12.1b NO, what is the website of the app commissioner?	Link
12.1e	If 12.1b NO, what is the app commissioner's funding model?	Pick from list
12.2a	What is the app copyright statement?	Text
12.2b	What is the marketplace copyright statement?	Text
12.3a	Does the app include adverts?	Yes/No
12.3b	What format adverts are used?	Pick from list
12.3c	If 12.3a YES, does the app publisher have an advertising policy?	Yes/No
12.3d	If 12.3b YES, provide details.	Text
12.4a	Does the app offer paid-for enhancements?	Yes/No
12.4b	If 12.4a YES, what is the nature of these enhancements?	Text
12.4c	If 12.4a YES, is the app functional (for asthma) without the enhancements?	Yes/No
12.4d	If 12.4a YES, what is the cost of the necessary enhancements?	£
12.5a	Is there a clinical disclaimer in the app?	Yes/No
12.5b	If 12.5a YES, quote appropriate text from disclaimer	Text
12.5a	Is there a general software disclaimer in the app?	Yes/No
12.5b	If 12.5a YES, quote appropriate text from disclaimer	Text
12.6a	Is the app accredited or endorsed by any third party (distinct from the app commissioner)?	Yes/No
12.6b	If 12.6a YES, provide details	Text
12.7a	Does the app offer a local help system?	Yes/No
12.7b	Does the app offer online help system?	Yes/No
12.8a	Is there a support contact?	Yes/No
12.8b	If 12.8a YES, is there a support website?	Yes/No
12.8c	If 12.8b YES, what is the support website link?	Link
12.8d	If 12.8a YES, is there a support email?	Email Address
12.8e	If 12.8a YES, provide any other details	Text
Quality assurance		
13.1a	Were any textual/grammatical errors found in written or multimedia informational content?	Yes/No
13.1b	If 13.1a YES, provide relevant example(s)	Text
13.2a	Were any textual/grammatical errors found in the user interface?	Yes/No
13.2b	If 13.2a YES, provide relevant example(s)	Text
13.3a	Were any software errors encountered during test use?	Yes/No
13.3b	If 13.3a YES, provide details	Text
13.4a	Were any network/resource errors encountered during use?	Yes/No
13.4b	If 13.4a YES, provide details	Text
13.5a	Does the app appropriately validate data entry?	Yes/No
13.5b	If 13.5a YES or NO, provide details.	Text
13.6	Any other problems/issues identified?	Text
Other		
13.7	Notes	Text

Table AF6

Operational criteria for assessing inhaler technique education

Criteria covering eight domains. All criteria are sourced from their respective product literature[5-12] except where otherwise cited. Emboldened items are those necessary for delivery of drug into the lungs, extending previously developed criteria[13] with three further ‘critical’ issues[14]: removal of any mouthpiece cover; avoiding inhalation against closed teeth or through nose rather than mouth.

Domain	Criteria
Inhaler preparation	▪ ***All inhalers***<br>o **Mouthpiece cover removal** [14]<br>▪ *Accuhaler*<br>o **Blister pack loading**<br>o **Preparation by opening mouthpiece using thumb grip and then sliding lever until click heard**<br>▪ *Autohaler*<br>o **Preparation by raising lever on inhaler top**<br>▪ *Clickhaler, Easyhaler*<br>o 3-5 vigorous vertical shakes<br>o **Preparation by pressing inhaler top once only until click heard**, **then releasing**<br>▪ *Diskhaler*<br>o **Blister pack loading**<br>o **Preparation by sliding tray in and out and opening lid to pierce blister**<br>▪ *Easi-breathe inhaler*<br>o Vertical grasp not covering vents.<br>▪ *Handihaler*<br>o **Blister insertion and piercing** without shaking<br>▪ *Pressurised Metered Dose Inhaler (pMDI)*<br>o **Preparation by 4-5 shakes** (Justified by [15])<br>▪ *pMDI with spacer*<br>o As for pMDI, and:<br>o **Spacer mouthpiece cover removal**<br>o pMDI fitted firmly into compatible spacer<br>▪ *Turbohaler*<br>o **Preparation by turning base grip as far as possible in one direction and then the other until click heard**
Inhaler positioning	▪ ***All inhalers***<br>o Comfortable but complete exhalation, not through inhaler<br>o Sitting or standing position<br>o Appropriate inhaler grasp<br>o **Positioning inhaler/spacer mouthpiece in mouth and sealing lips without biting** (or in front of mouth for open mouth pMDI technique) [14]<br>▪ *Diskhaler*<br>o Mouth seal not covering vents on mouthpiece.
Inhalation/actuation method	▪ ***All inhalers***<br>o **Inward breath through mouth not nose** [14]<br>▪ *Accuhaler, Clickhaler, Handihaler*<br>o **Steady and deep inward breath** (no need for manual actuation)<br>▪ *Autohaler, Easi-Breathe Inhaler*

<table>
<tr><th>Domain</th><th>Criteria</th></tr>
<tr><td></td><td>
Slow, deep inward breath that continues when actuation click is heard (no need for manual actuation).
Diskhaler, Easyhaler, Turbohaler
Inward breath as deeply and as forcefully as possible (no need for manual actuation)
pMDI
Steady, deep inward breath
Manual actuation just after inhalation starts and well before inhalation ends
Inhalation not interrupted by actuation
pMDI with spacer
Single actuation into spacer followed immediately by steady deep inward breath or single actuation into large volume spacer during normal breathing.
</td></tr>
<tr><td>Breath holding and exhalation sequence</td><td>
All inhalers
Inhaler removed from mouth during breath holding
No exhalation through inhaler
Accuhaler, Autohaler, Easi-breathe inhaler
Breath held for 10 seconds then slow exhalation
Clickhaler, Diskhaler, Easyhaler, pMDI
Breath held for at least 5 seconds or as long as comfortable.
pMDI with spacer
As for pMDI after removing spacer from mouth or continued tidal breathing for 5 breaths with exhalation/inhalation into spacer
Turbohaler
Gentle exhalation after removing inhaler from mouth
</td></tr>
<tr><td>Timing of additional dose(s)</td><td>
Accuhaler
Repeated immediately if needed by sliding thumb grip to original position, listening for click
Autohaler
Repeated immediately if needed by lowering then raising lever on inhaler top
Clickhaler, Handihaler, Turbohaler
Repeated immediately if needed
Diskhaler
Repeated immediately if needed by repeating tray sliding method, opening blister only when needed
Easi-breathe Inhaler, Easyhaler
Delay one minute prior to repetition, cap closed between repetitions
pMDI, pMDI with spacer
Delay 30 seconds prior to repetition
</td></tr>
<tr><td>Mouth rinsing</td><td>
All inhalers
Rinsing and spitting after steroid use to reduce risk of candidiasis [2, 3] or to remove taste
</td></tr>
<tr><td>Identifying empty inhaler</td><td>
All inhalers
Cannot be judged by sounds made when inhaler shaken
Accuhaler, Diskhaler
Dose counter showing 0 indicating need to replace blister pack
Autohaler, Easi-Breathe inhaler
Empty when no propellant discharged during use
Clickhaler
</td></tr>
</table>

Domain	Criteria
	o Dose counter background turns red when ten doses left and again when no doses remain<br>▪ *Easyhaler*<br>o Dose counter numbers turn red when 20 doses left; cannot be judged by powder level seen in inhaler window<br>▪ *Handihaler*<br>o Single capsule emptied after each inhalation sequence<br>▪ *pMDI*<br>o Relies on tracking total number of doses stated on label<br>▪ *Turbohaler*<br>o Appearance of red mark in dose indicator window when approximately 20 doses left
Inhaler care	▪ ***All inhalers***<br>o By replacing cover after use<br>▪ *Autohaler*<br>o By wiping mouthpiece with dry tissue<br>▪ *Diskhaler*<br>o Using supplied brush to remove excess powder<br>▪ *Clickhaler, Easi-breathe inhaler, Easyhaler, Turbohaler*<br>o By wiping mouthpiece with dry tissue only; immersing no part in water<br>▪ *Handihaler*<br>o Washing without detergent and air drying per manufacturer instructions<br>▪ *pMDI*<br>o Risk of blockage preventing fine spray addressed by removing canister and rinsing plastic casing weekly<br>▪ *Spacer*<br>o By washing in detergent and air drying per manufacturer instructions [1, 2]

References

1. British Thoracic Society, Scottish Intercollegiate Guidelines Network: *British guideline on the management of asthma - a national clinical guideline. May 2008, revised January 2012*. Edinburgh, Scotland: SIGN; 2012.
2. National Asthma Education and Prevention Program: *Expert panel report 3: guidelines for the diagnosis and management of asthma. Full report 2007*. Bethseda, MD, USA: National Institutes of Health; 2007.
3. Global Initiative for Asthma: **Global strategy for asthma management and prevention. Updated 2012** [http://www.ginasthma.org]
4. Huckvale K, Car M, Morrison C, Car J: **Apps for asthma self-management: a systematic assessment of content and tools**. *BMC Med* 2012, **10**(1):144.
5. Allen & Hanburys Limited: *Seretide 100, 250, 500 accuhaler. Package leaflet: information for the user.* . Uxbridge, United Kingdom: Glaxo Wellcome UK Limited; 2014.
6. Glaxo Wellcome UK Limited: *Serevent diskhaler 50 microgram per dose inhalation powder. Package leaflet: information for the user*. Uxbridge, United Kingdom: Glaxo Wellcome UK Limited; 2014.
7. GlaxoSmithKline UK: *Ventolin evohaler. Package leaflet: information for the user*. Uxbridge, United Kingdom: Glaxo Wellcome UK Limited; 2014.
8. Teva UK Limited: *QVar 50 and 100 autohaler beclometasone dipropionate. Package leaflet: information for the user*. Eastbourne, United Kingdom: Teva UK Limited; 2012.
9. Teva UK Limited: *QVar easi-breathe 50 micrograms per actuation inhaler beclometasone dipropionate. Package leaflet: information for the user*. Eastbourne, United Kingdom: Teva UK Limited; 2012.
10. Orion Pharma (UK) Limited: *Easyhaler salbutamol sulphate 100 and 200 micrograms/dose inhalation powder. Package leaflet: information for the user*. Espoo, Finland: Orion Corporation; 2013.
11. RPH Pharmaceuticals AB: *Asmasal clickhaler. Inhalation powder 95 micrograms/inhalation. Salbutamol (as sulphate). Patient information leaflet*. Jorbro, Sweden: RPH Pharmaceuticals AB; 2009.
12. AstraZeneca UK Limited: *Bricanyl turbohaler, 0.5mg/dose inhalation powder. Package leaflet: information for the user*. Luton, United Kingdom: AstraZeneca UK Limited; 2012.
13. van der Palen J, Klein JJ, Kerkhoff AHM, van Herwaarden CLA, Seydel ER: **Evaluation of the long-term effectiveness of three instruction modes for inhaling medicines**. *Patient Educ Couns* 1997, **32, Supplement 1**(0):S87-S95.
14. Melani AS, Bonavia M, Cilenti V, Cinti C, Lodi M, Martucci P, Serra M, Scichilone N, Sestini P, Aliani M *et al*: **Inhaler mishandling remains common in real life and is associated with reduced disease control**. *Respir Med* 2011, **105**(6):930-938.
15. Everard ML, Devadason SG, Summers QA, Le Souëf PN: **Factors affecting total and "respirable" dose delivered by a salbutamol metered dose inhaler**. *Thorax* 1995, **50**(7):746-749.
16. Health on the Net Foundation: **Operational definition of the HONcode principles. Guidelines v2.0** [http://www.hon.ch/HONcode/Guidelines/guidelines.html#Review]
